# Depressive symptoms and cognitive impairment among rural Chinese older women: anxiety and poor sleep as sequential correlates—A cross-sectional analysis

**DOI:** 10.3389/fpubh.2026.1797904

**Published:** 2026-05-08

**Authors:** Hongli Liu, Ping Ju, Jin Yang, Shuqiao Wang, Ling Li, Yuting Xiao, Yuhang Zhu, Jing Jiang, Xiaoling Tang

**Affiliations:** 1 Department of Critical Care Medicine, Chongqing Academy of Medical Sciences, Chongqing General Hospital, Chongqing University, Chongqing, China; 2 Nursing School of Zunyi Medical University, Zunyi, China; 3 Department of Respiratory and Critical Care Medicine, Affiliated Hospital of North Sichuan Medical College, Nanchong, China; 4 Key Laboratory of Brain Function and Brain Disease Prevention and Treatment of Guizhou Province, Affiliated Hospital of Zunyi Medical University, Zunyi, China

**Keywords:** anxiety, cognitive impairment, depressive symptoms, older women, sleep quality

## Abstract

**Background:**

Depressive symptoms are a well-established predictor of late-life cognitive impairment. Furthermore, anxiety and sleep quality—both correlates of cognitive impairment—could mediate the depression–cognition association in older women. Nevertheless, limited research has empirically tested this hypothesis using causal mediation analysis.

**Methods:**

This cross-sectional study recruited 718 older women from rural areas of Sichuan, Chongqing, and Guizhou provinces in China using convenience sampling. Trained assessors administered the Patient Health Questionnaire-9, the Chinese Version of the Montreal Cognitive Assessment, the Generalized Anxiety Disorder-7 Scale, and the Pittsburgh Sleep Quality Index to assess depressive symptom severity, cognitive performance, anxiety levels, and sleep quality, respectively. Statistical analyses were performed using SPSS 29.0, with chi-square testing, Spearman rank correlation, and bootstrapped mediation analysis (5,000 resamples, bias-corrected) executed through the PROCESS macro (version 4.1).

**Results:**

Both direct and indirect effects of depressive symptoms on cognitive impairment were found in older women in rural areas (*p* < 0.05). As predicted, anxiety and sleep quality mediated the relationship between depressive symptoms and cognitive impairment [*B*_Anxiety_ = −0.580, 95% confidence interval (CI): −0.737 to −0.420; *B*_sleep quality_ = −0.168, 95% CI: −0.239 to −0.101]. In addition, serial mediation analyses indicated that the association of depressive symptoms and cognitive impairment was mediated by anxiety and sleep quality in a sequential manner (*B* = −0.059, 95% CI: −0.122 to −0.008).

**Conclusion:**

This study identified a modifiable pathway in older rural Chinese women: anxiety and sleep quality sequentially mediated the link between depressive symptoms and cognitive impairment, with anxiety showing an association with sleep disturbances that correlated with greater impairment. Multicomponent strategies addressing this symptom triad suggest intervention targets to mitigate the risk of decline in this underserved population.

## 1 Introduction

A meta-analysis indicated that cognitive impairment affects 23.2% of rural-dwelling older women (1), with 10–15% progressing to dementia annually (2). According to the World Health Organization, women account for roughly two-thirds of all dementia and Alzheimer’s disease (AD) cases worldwide, and this proportion is projected to rise (3). Consequently, the disproportionate burden of cognitive impairment in older women constitutes a major global public health challenge, entailing higher morbidity, poorer health outcomes, and increased mortality (4, 5). Although no definitive cure exists, mounting evidence indicates that cognitive decline can be delayed—if not partially reversed—through timely, evidence-based interventions targeting modifiable risk factors (6). Identifying the factors and mechanisms that underlie cognitive impairment in this population is therefore imperative.

Among solitary-living seniors, 47.8% experience depressive symptoms (7), with countryside residents 1.37 times as likely to be affected as their city-dwelling peers (8). Those with depression show a significantly elevated risk of mild cognitive impairment [relative risk = 1.49, 95% confidence interval (CI): 1.13–1.86] (9). Anxiety confers an approximately 50% increased risk of AD, while sleep disorders, frequently comorbid with anxiety/depression, may accelerate the development of cognitive impairment (10). Notably, older women who self-report ≤6 h of sleep show a 1.29-fold higher prevalence of cognitive impairment than those sleeping >6–8 h (11). Multidomain cognitive impairment—spanning attention, working memory, language acquisition, and visual learning—co-occurs with depression, anxiety, and sleep disturbances (12–15). One study (16) found that patients with AD often develop comorbid psychiatric symptoms—including sleep disturbances, anxiety, and depression—in the advanced stages of the illness, and that these disturbances have complex bidirectional interactions with neurodegeneration. The study indicated that AD prevalence rises sharply with age, from 3.4% in individuals aged 65–74 years to 13.8% at 75–84 years and 35.8% at ≥85 years (17). However, aging *per se* is not pathognomonic of AD; rather, it interacts with modifiable psychosocial stressors to potentiate neurocognitive vulnerability.

While depression–cognition associations are well-documented, mechanistic pathways—particularly the depression–anxiety–sleep nexus—remain elusive in underserved rural populations. This evidence gap is pronounced among aging women in rural China. We therefore executed a large-scale cross-sectional investigation across southwestern China to elucidate these interrelationships. The results will inform culturally adapted, scalable frameworks to mitigate affective distress and protect neurocognitive function in this vulnerable demographic.

## 2 Method

### 2.1 Participants

A cross-sectional survey was conducted from January to October 2024 in three southwestern Chinese regions: Sichuan Province, Chongqing Municipality, and Guizhou Province. With the support of local health authorities, eligible older women were invited through community announcements, telephone calls, and when necessary, door-to-door visits. Upon written informed consent, trained personnel delivered structured interviews in confidential settings, with participant anonymity and data security ensured. Regional coverage was optimized via a convenience sampling design. The Medical Ethics Committee of Chongqing General Hospital (KYS2024-017-01) oversaw the study, with all procedures conforming to the Declaration of Helsinki standards.

#### 2.1.1 Inclusion criteria

1. Female sex, aged 60 years and older;
2. Residing in rural areas of China for ≥1 year;
3. Willing to participate and able to provide informed consent;
4. Capable of completing cognitive assessments (e.g., able to communicate in the local dialect).

#### 2.1.2 Exclusion criteria

1. Severe diseases affecting major organ systems (e.g., heart, brain, lungs, kidneys);
2. Diagnosed with severe psychiatric disorders or significant sensory or communication impairments that prevent valid assessment.

#### 2.1.3 Additional exclusion criteria

1. >20% missing data on key study variables;
2. Inconsistent or patterned questionnaire responses suggestive of unreliable data.

### 2.2 Sample size

According to the formula $N=\frac{Z_{\alpha \;P(1-P)}^{2}}{\delta^{2}}$ , meta-analytic data place cognitive impairment prevalence at 23.2% among older Chinese women (1). Based on this estimate, the margin of error was set at 15% of the overall prevalence (18). Applying the standard sample size formula yielded a required sample of 566. To account for a projected 20% attrition rate, the recruitment target was raised to 708, ensuring adequate statistical power.

## 3 Measures

### 3.1 General Information Questionnaire

A structured questionnaire was developed for rural Chinese older women, comprising two sections: sociodemographic characteristics (age, ethnicity, education) and health-related variables (chronic conditions, surgical history, tobacco and alcohol use). Trained staff obtained standardized anthropometric measurements. Using World Health Organization body mass index (BMI) cutoffs, participants were classified as underweight (<18.5 kg/m^2^), normal weight (18.5–24.9 kg/m^2^), overweight (25.0–29.9 kg/m^2^), or obese (≥30.0 kg/m^2^) (19).

### 3.2 Montreal Cognitive Assessment

Assessment of cognitive function: The Montreal Cognitive Assessment (MoCA), widely used for cognitive screening, was employed. Developed in Canada by Nasreddine et al. (20), it adapts Mini-Mental State Examination items and scoring based on clinical experience. Initially cross-culturally adapted for Chinese populations by Wang et al. (21), the scale was formally implemented in China’s clinical practice that year. The Chinese version of the MoCA is adapted for older adults with low education levels and demonstrates no ceiling effect in highly educated elders. The MoCA yields a 30-point total, with scores below 26 indicating cognitive impairment.

### 3.3 Patient Health Questionnaire-9

The Patient Health Questionnaire-9 (PHQ-9), a self-report measure, captures depressive symptom presence and severity over the prior 2 weeks (22). Developed from Diagnostic and Statistical Manual of Mental Disorders, 5th Edition criteria, this nine-item instrument yields total scores of 0–27, with elevated values indicating greater symptom burden. Meta-analytic validation of the Chinese adaptation demonstrated sensitivity of 0.88 (95% CI: 0.85–0.91) and specificity of 0.89 (95% CI: 0.82–0.94) (23), supporting its utility as a depression screening tool.

### 3.4 Generalized Anxiety Disorder Scale-7

Anxiety levels were assessed using the Generalized Anxiety Disorder Scale-7 (GAD-7), a standardized tool developed by Spitzer et al. (24) for screening and evaluating generalized anxiety disorder. This scale has been demonstrated to be a reliable and valid self-report instrument (25). Over the prior 2-week period, respondents indicated how often and how intensely they experienced each anxiety symptom using a 4-point (range: 0–3) Likert scale. Aggregate scores span 0–21, where higher scores correspond to heightened anxiety levels.

### 3.5 Pittsburgh Sleep Quality Index

Developed by Buysse et al. (26) in 1989, the Pittsburgh Sleep Quality Index (PSQI) is a widely used measure of sleep quality over the prior month. This 19-item instrument assesses seven domains: subjective sleep quality, sleep latency, duration, efficiency, disturbances, medication use, and daytime dysfunction. The Chinese adaptation by Liu et al. (27) demonstrated a Cronbach’s *α* of 0.852 and test–retest reliability of 0.829.

### 3.6 Research team composition and quality control

The research team was composed of two senior instructors, one intermediate nurse, four junior nurses, and one social worker recruited from the collection site. Prior to the survey, all participants underwent specialized training and completed a simulation practice assessment. Those who did not pass the initial assessment received additional training until they consistently met the data collection standards.

## 4 Data analysis

After dual independent data entry into Excel, discrepancies were reconciled. Analytical procedures were conducted using SPSS 29.0 and the PROCESS macro (version 4.1, Model 6). Categorical variables are presented as counts and percentages, and continuous measures as [median (interquartile range, IQR)] following Shapiro–Wilk assessment of the data distribution. Skewed outcomes were evaluated by the Mann–Whitney *U* test, and nominal variables by chi-square analysis. Bivariate linkages among cognition, depression, anxiety, and sleep were assessed using Pearson correlation analysis. Serial mediation utilized 5,000 bootstrap samples to derive 95% CIs for indirect pathways, with effects deemed significant when the CIs excluded zero. A two-sided *α* of 0.05 was used in all inferential tests.

## 5 Results

### 5.1 Participant characteristics

**Figure 1:**
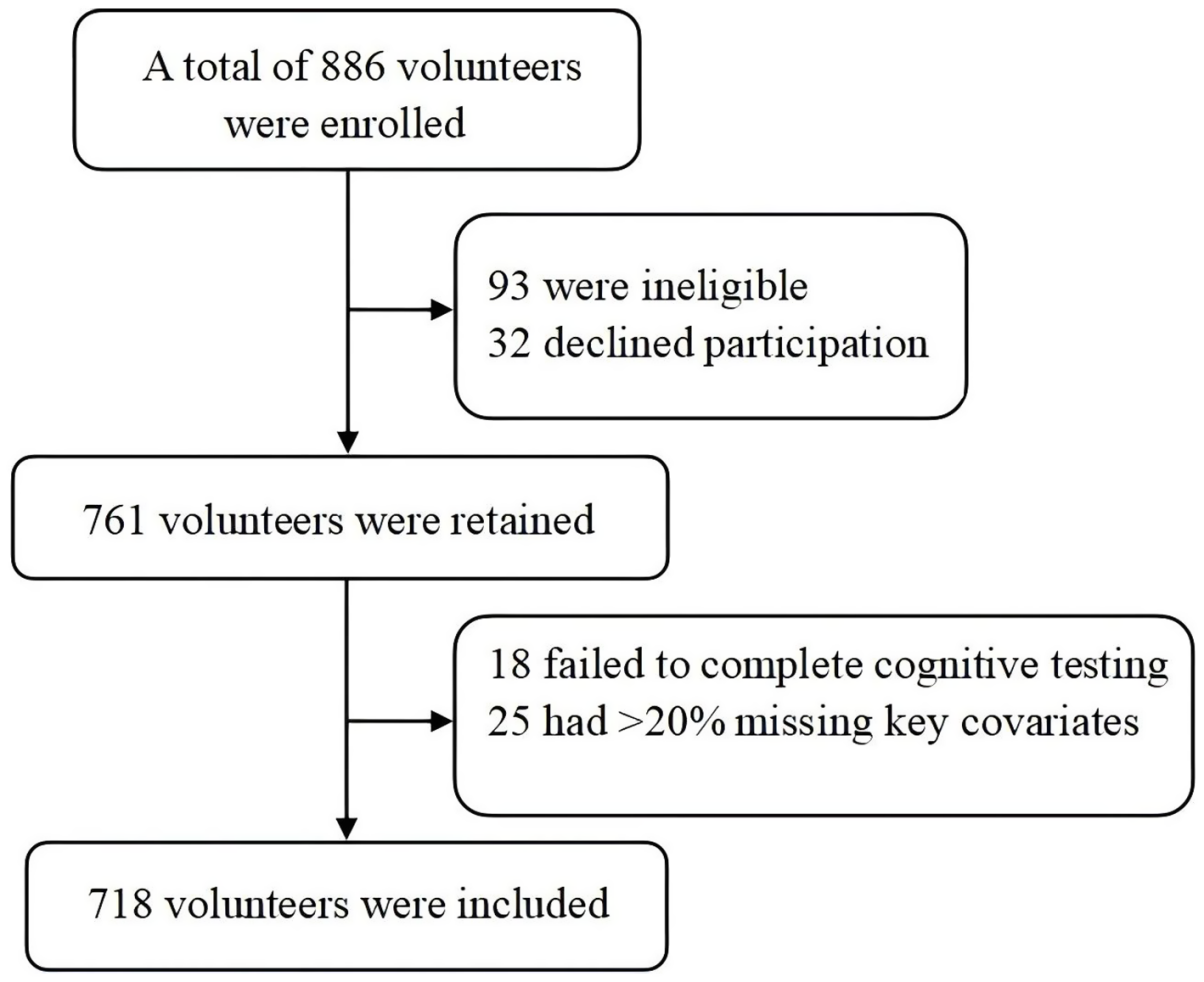
Flowchart of participant recruitment.

Of the 866 older rural women initially approached, 718 met the inclusion criteria and were retained for the analysis (response rate: 82.9%). Participants could withdraw at will. The recruitment flow is detailed in Figure 1. The sample comprised 718 older women in rural areas, of whom 253 had cognitive impairment (prevalence: 35.2%). The impaired group was significantly older. Nearly half (45%) had no formal schooling, two-thirds (65.9%) had a normal BMI, and one-third (31.2%) had multimorbidity. The groups differed significantly in age, marital status, living arrangements, education, chronic disease burden, and surgical history (*p* < 0.05; Table 1). Impairment severity varied across the MoCA, PHQ-9, GAD-7, and PSQI scores (*p* < 0.05).

**Table 1:** Demographic characteristics of the participants.

Variables	Total (*n* = 718)	Cognitive impairment		*x*^2^/*Z*	*P*
		No (*n* = 465)	Yes (*n* = 253)		
Age groups					
60–69 years	110 (15.32%)	85 (18.28%)	25 (9.88%)	129.409^a^	<0.001
70–79 years	507 (70.61%)	365 (78.49%)	142 (56.12%)		
≥80 years	101 (14.07%)	15 (3.23%)	86 (34.00%)		
BMI				3.487^a^	0.322
Normal weight	473 (65.9%)	317 (68.2%)	156 (61.7%)		
Underweight	75 (10.4%)	45 (9.7%)	30 (11.9%)		
Overweight	149 (20.8%)	89 (19.1%)	60 (23.7%)		
Obese	21 (2.9%)	14 (3.0%)	7 (2.8%)		
Ethnic groups				1.768^a^	0.184
Han nationality	616 (85.8%)	393 (84.5%)	223 (88.1%)		
Ethnic minorities	102 (14.2%)	72 (15.5%)	30 (11.9%)		
Marital status				25.096^a^	<0.001
Spouses	563 (78.4%)	391 (84.1%)	172 (68%)		
No spouse	155 (21.6%)	74 (15.9%)	81 (32.0%)		
Living alone				23.285^a^	<0.001
Yes	182 (25.3%)	91 (19.6%)	91 (36.0%)		
No	536 (74.7%)	374 (80.4%)	162 (64.0%)		
Education				6.325^a^	0.042
Illiterate	323 (45.0%)	194 (41.7%)	129 (51.0%)		
Elementary school	157 (21.9%)	104 (22.4)	53 (20.9%)		
Middle school and above	238 (33.1%)	167 (35.9%)	71 (28.1%)		
Chronic diseases				11.205^a^	0.011
0	220 (30.6%)	160 (34.4%)	60 (23.7%)		
1	274 (38.2%)	176 (37.8%)	98 (38.7%)		
2	122 (17.0%)	70 (15.1%)	52 (20.6%)		
>2	102 (14.2%)	59 (12.7%)	43 (17.0%)		
Surgery history				3.981^a^	0.046
No	430 (59.9%)	291 (62.6%)	139 (54.9%)		
Yes	288 (40.1%)	174 (37.4%)	114 (45.1%)		
Smoking history				0.001^b^	0.979
No	711 (99.0%)	461 (99.1%)	250 (98.8%)		
Yes	7 (1.0%)	4 (0.9%)	3 (1.2%)		
Drinking history				0.071^a^	0.790
No	676 (94.2%)	437 (94.0%)	239 (94.5%)		
Yes	42 (5.8%)	28 (6.0%)	14 (5.5%)		
**MoCA score**	26 (19, 26)	26 (26, 27)	17 (15, 19)	**−23.33^c^**	**<0.001**
**PHQ-9 score**	3 (2, 6)	2 (2, 3)	7 (6, 8)	**−17.91^c^**	**<0.001**
**GAD-7 score**	2 (1, 5)	2 (1, 2)	6 (5, 6)	**−18.45^c^**	**<0.001**
**PSQI score**	8 (6, 11)	7 (5, 9)	11 (9, 14)	**−13.93^c^**	**<0.001**

^a^Chi-square test; ^b^Corrected chi-square test; ^c^Mann–Whitney U test. BMI, body mass index; MoCA, Montreal Cognitive Assessment; PHQ-9, Patient Health Questionnaire-9; GAD-7, Generalized Anxiety Disorder Scale-7; PSQI, Pittsburgh Sleep Quality Index.

### 5.2 Factors associated with cognitive function in rural older women: multivariate logistic regression analysis

A logistic regression analysis was conducted to investigate how multiple factors influence cognitive function in rural older women (Table 2). The following variables were controlled for: lives alone (no = 0, yes = 1), surgery (no = 0, yes = 1), age (60–69 years = 0, 70–79 years = 1, ≥80 years = 2), education (illiterate = 0, primary = 1, ≥ junior middle school = 2), and chronic disease count (no = 0, one chronic disease = 1, two chronic diseases = 2, >2 chronic diseases = 3). Multivariate logistic regression analysis revealed that age, education level, number of chronic diseases, depressive symptoms, anxiety, and sleep quality were significantly associated with cognitive impairment in rural older women (*p* < 0.05).

**Table 2:** Factors associated with cognitive function in rural older women: multivariate logistic regression analysis.

Variable	*B*	SE	*P*	OR (95% CI)
Age groups				
70–79 years	0.251	0.415	0.545	1.286 (0.570, 2.901)
≥80 years	2.222	0.553	<0.001	9.223 (3.120, 27.260)
Education				
Elementary	0.418	0.356	0.240	1.519 (0.756, 3.052)
Middle school and above	1.506	0.353	<0.001	4.508 (2.256, 9.009)
Chronic diseases				
1	0.149	0.336	0.658	1.16 (0.601, 2.241)
2	0.932	0.404	0.021	2.539 (1.15, 5.608)
>2	−0.172	0.462	0.71	0.842 (0.341, 2.081)
PHQ-9 score	0.212	0.107	0.047	1.236 (1.003, 1.523)
GAD-7 score	0.789	0.129	<0.001	2.20 (1.709, 2.833)
PSQI score	0.283	0.053	<0.001	1.327 (1.195, 1.473)
*F*	381.297			
*R^2^*	0.737			
*∆R^2^*	0.052			

PHQ-9, Patient Health Questionnaire-9; GAD-7, Generalized Anxiety Disorder Scale-7; PSQI, Pittsburgh Sleep Quality Index; SE, standard error; OR, odds ratio; CI, confidence interval.

### 5.3 Correlational analysis of variables

**Figure 2:**
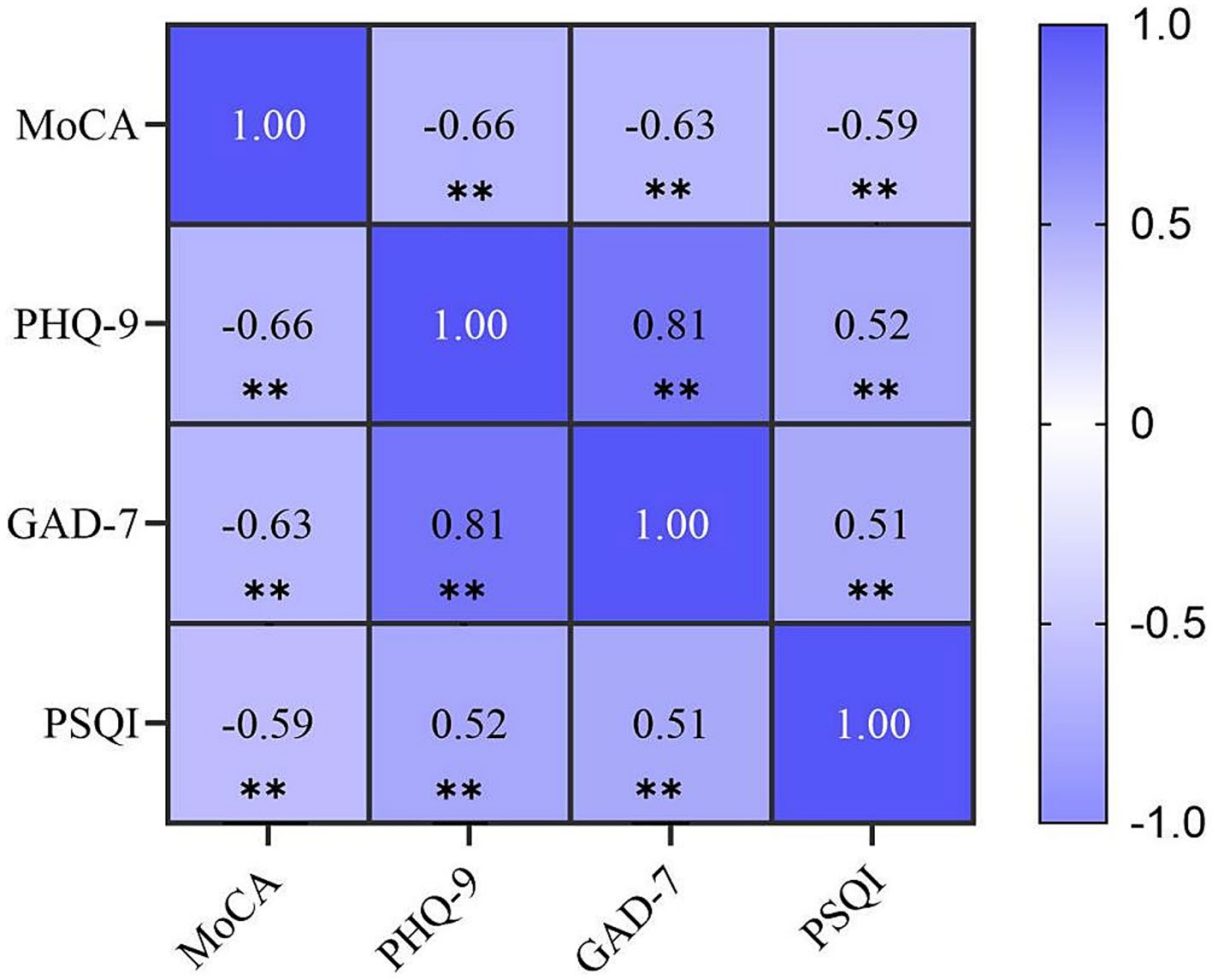
Pearson correlation matrix of the study variables. *p* < 0.001. PHQ-9, Patient Health Questionnaire-9; GAD-7, Generalized Anxiety Disorder Scale-7; PSQI, Pittsburgh Sleep Quality Index; MoCA, Montreal Cognitive Assessment.

The correlations of all four main variables are shown in Figure 2. Cognitive function was negatively correlated with depressive symptoms (*r* = −0.66, *p* < 0.01), anxiety (*r* = −0.63, *p* < 0.01), and sleep quality (*r* = −0.59, *p* < 0.01). Depressive symptoms were strongly positively correlated with anxiety (*r* = 0.81, *p* < 0.01) and moderately correlated with sleep quality (*r* = 0.52, *p* < 0.01). The GAD-7 score was also positively correlated with sleep quality (*r* = 0.51, *p* < 0.01).

### 5.4 Verification of the chain mediation model

A hierarchical regression analysis was conducted using PROCESS Model 6 (version 4.1), with age, education level, and number of chronic diseases included as covariates. The results are presented in Table 3. As shown in Table 3, depressive symptoms were positively associated with anxiety (*β* = 0.752, 95% CI: 0.716–0.788) and sleep quality (*β* = 0.483, 95% CI: 0.328–0.638). Anxiety was positively associated with sleep quality (*β* = 0.228, 95% CI: 0.053–0.403). After accounting for covariates and mediators, depressive symptoms remained negatively associated with cognitive function (*β* = −0.523, 95% CI: −0.711 to −0.334), while both anxiety (*β* = −0.772, 95% CI: −0.980 to −0.564) and sleep quality (*β* = −0.347, 95% CI: −0.433 to −0.261) were independently associated with cognitive function.

**Table 3:** Hierarchical regression analysis of the sequential mediation model with covariates.

Variable	Model 1 (GAD-7)		Model 2 (PSQI)		Model 3 (MoCA)	
	Effect size	Standard error	Effect size	Standard error	Effect size	Standard error
Control variables						
Age	0.237**	0.079	0.456*	0.191	0.931***	0.226
Education level	0.014	0.048	−0.740***	0.114	0.516***	0.139
Chronic diseases	−0.012	0.041	0.230*	0.097	−0.289*	0.115
Independent variable						
PHQ-9 score	0.752***	0.018	0.483***	0.079	−0.523***	0.096
Mediating variable						
GAD-7 score			0.228*	0.089	−0.772***	0.106
PSQI score					−0.347***	0.044

PHQ-9, Patient Health Questionnaire-9; GAD-7, Generalized Anxiety Disorder Scale-7; PSQI, Pittsburgh Sleep Quality Index; MoCA, Montreal Cognitive Assessment; *P < 0.05; **P < 0.01; ***P < 0.001.

This study then examined the chain mediation effects among depressive symptoms, anxiety, sleep quality, and cognitive function in rural older women (Table 4). The results indicated that the 95% CIs for all pathways excluded zero. Depression was significantly and negatively associated with cognitive impairment (*β* = −0.523, 95% CI: −0.711 to −0.334), with this direct effect accounting for 39.32% of the total effect. The total indirect effect was estimated as −0.807, representing 60.68% of the total effect. Further decomposition revealed three specific indirect pathways: mediation through anxiety alone, mediation through sleep quality alone, and a sequential chain involving all three constructs (depression → anxiety → sleep quality → cognitive impairment). The 95% CIs for these three indirect paths also excluded zero, confirming their statistical significance. Individually, anxiety and sleep quality mediated 43.61 and 12.63% of the total effect, respectively. Additionally, the chain mediation pathway from depression through anxiety and subsequently sleep quality to cognitive impairment accounted for 4.44% of the total effect.

**Table 4:** Analysis of chain mediation effects in older women in rural areas.

Path	Effect value	Boost standard error	Boost LLCI	Boost ULCI	Effect proportion
Total effect	−1.330	0.054	−1.436	−1.224	100%
Direct effect	−0.523	0.096	−0.711	−0.334	39.32%
Total indirect effect	−0.807	0.083	−0.972	−0.642	60.68%
Path 1^a^	−0.580	0.081	−0.737	−0.420	43.61%
Path 2^b^	−0.168	0.036	−0.239	−0.101	12.63%
Path 3^c^	−0.059	0.029	−0.122	−0.008	4.44%

^a^PHQ-9–GAD-7–MoCA; ^b^PHQ-9–PSQI–MoCA; ^c^PHQ-9–GAD-7–PSQI–MoCA.

### 5.5 Multiple mediation analyses

**Figure 3:**
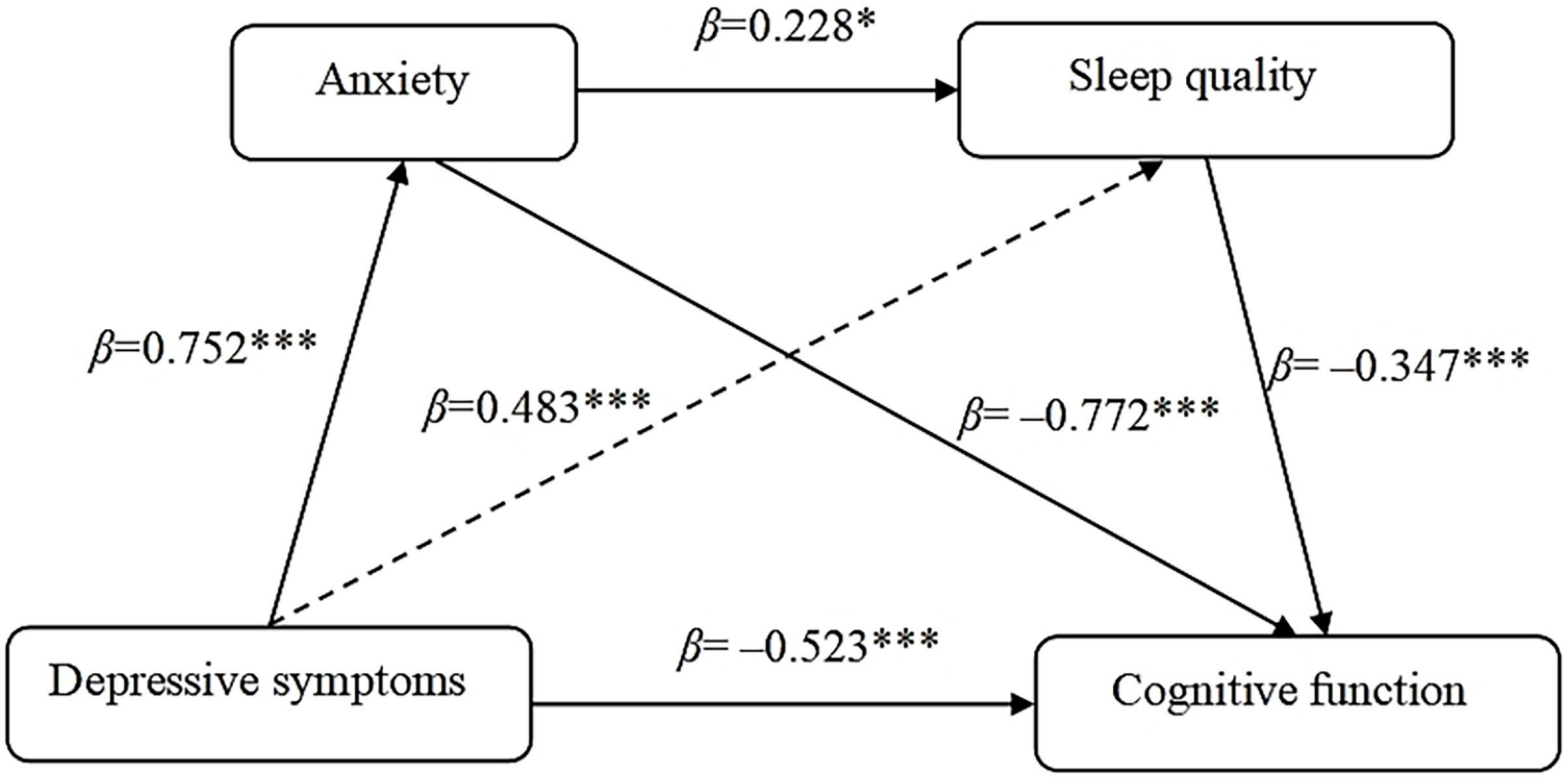
Chain mediation model and the *β* of each path. *p < 0.05; ***p < 0.001.

A multiple mediation model was constructed to examine how anxiety and sleep quality mediate the effect of depressive symptoms on cognitive function. In this model, depressive symptoms served as the independent variable, cognitive function as the dependent variable, and anxiety and sleep quality as parallel mediators. Age, education, residence, and other demographic factors were statistically controlled. As illustrated in Figure 3, depressive symptoms had a significant negative direct effect on cognitive function (path coefficient = −0.523). Additionally, depressive symptoms were positively associated with both anxiety and sleep quality. In turn, these two mediators exhibited negative associations with cognitive function. Notably, anxiety and sleep quality were positively intercorrelated.

## 6 Discussion

This study examined interrelationships among depressive symptoms, anxiety, sleep quality, and cognitive function. Correlation analyses revealed significant pairwise associations among these variables. Subsequent mediation modeling identified two distinct indirect pathways linking depressive symptoms to cognitive function: one mediated by anxiety and the other by sleep quality. Specifically, depressive symptoms influenced cognitive function through anxiety or sleep quality independently. Furthermore, a serial mediation pathway emerged wherein depressive symptoms were associated with cognitive function sequentially through anxiety and sleep quality, underscoring the importance of considering the interplay between these two mediators when examining depression–cognition associations.

Among rural older women, this mechanism unfolds as follows: limited access to filial support and reduced social engagement foster social isolation, precipitating depressive symptoms and accelerating cognitive decline. These symptoms, in turn, intensify anxiety—manifesting as health-related worries, financial insecurity, loneliness, and a diminished sense of purpose—which further disrupts sleep quality. Chronic sleep disturbances not only exacerbate depression but also form a feedback loop that amplifies cognitive deterioration. This underscores the centrality of psychosocial factors in shaping mental health trajectories in this vulnerable population.

### 6.1 Analysis of the current situation regarding the cognitive function and depressive symptoms of older women in rural areas of China

The prevalence of cognitive impairment among rural women was 35.2%, which was higher than the 23.2% reported for older Chinese women in a recent meta-analysis (1). This elevation likely reflects our focus on rural women, who—being more often widowed and unwilling to burden their children—usually live alone. Limited mobility and the large physical distances between rural households foster loneliness and social isolation, both of which accelerate cognitive decline (28). Older adults living alone show faster deterioration in memory, attention, and executive function and score significantly lower on cognitive tests than their co-resident peers (29). Nevertheless, our estimate is lower than the 38.1% reported by Ren et al. (30) in rural older women; this discrepancy may be attributable to the exceptionally high prevalence of hypertension (79.7%) in their sample, a condition consistently identified as an independent predictor of cognitive decline (31, 32). Our study also revealed that ethnic minority older women had better cognitive function than Han women. As reported by Yue et al. (33), the prevalence of AD in Tibetans aged ≥60 years is significantly lower than in the Han population, potentially due to lifestyle, religious beliefs, and cultural factors influencing cognitive health. Additionally, international scholars such as Fadalla et al. (34) have identified racial/ethnic differences in cognitive function.

The prevalence of depressive symptoms among rural women aged ≥60 years in southwestern China was 36.4%, which is almost identical to the 37.8% (95% CI: 29.9, 45.9) reported for rural older adults in India (35). Almost half (45.0%) of our participants had never attended school; low educational attainment is a well-established correlate of increased depressive symptomatology (36). In addition, the marked estrogen and progesterone fluctuations that characterize the menopausal transition reduce serotonergic tone and alter *γ*-aminobutyric acid modulation, thereby elevating depression risk (37). In rural China, however, community-based pension and mental-health services remain scarce, and many women have lost traditional family support. The resultant social isolation further amplifies their vulnerability to depressive symptoms (38).

### 6.2 Role of depressive symptoms in cognitive impairment

Depressive symptoms demonstrated significant associations with compromised cognitive performance (*p* < 0.001), exhibiting a negative association. Aligning with prior investigations (39), advanced age was identified as a strong correlate of cognitive impairment, particularly among those aged ≥80 years, who exhibited a markedly increased risk (odds ratio = 9.223, 95% CI: 3.120–27.260). Among rural older adults, elevated depression severity corresponded to diminished cognitive capacity. Geriatric depressive manifestations have previously been implicated in expediting neurocognitive deterioration and elevating dementia susceptibility. Biologically, late-onset depression potentially expedites neurodegenerative processes through glucocorticoid-induced hippocampal volume reduction and upregulation of pro-inflammatory cytokines, consequently reducing the threshold for dementia onset (40). Within our rural sample, depressive symptoms exerted a robust direct influence on cognitive impairment, explaining 39.32% of the variance. This result substantiates that this association transcends mere comorbidity, constituting a salient risk trajectory toward cognitive decline. These observations highlight the imperative for tailored mental health interventions targeting older adults in under-resourced rural environments to impede the depression-to-dementia transition.

### 6.3 Mediating effect of anxiety

This investigation revealed that, relative to alternative indirect pathways, the serial mediation pathway traversing depressive symptoms and anxiety exerted the strongest effect on cognitive impairment. Consistent with prior research (41), elevated anxiety levels were associated with compromised cognitive performance; specifically, anxiety precipitated immediate memory deficits, whereas depression primarily disrupted delayed recall and executive functioning (42). The comorbid presentation of anxiety and depression consequently amplifies global cognitive impairment and elevates the risk for sustained cognitive deterioration. Additional evidence (43) indicates that anxiety symptoms, highly prevalent among older adults with depression, accelerate memory decline, further substantiating anxiety’s mediating role in the depression–cognition relationship. Clinical consensus also recognizes that cognitive impairment persists across all stages of depressive illness and engages in bidirectional interplay with anxiety: anxiety intensifies distractibility and undermines decision-making capacity, thereby perpetuating a self-reinforcing cycle of depressive symptoms → anxiety → cognitive deterioration (44). Current clinical guidelines advocate for multimodal treatment of comorbid anxiety and depression, integrating pharmacological agents with evidence-based non-pharmacological modalities such as psychotherapy, neuromodulation, and complementary interventions. Meta-analytic findings (45) further demonstrate that combined cognitive-behavioral therapy and pharmacotherapy outperforms pharmacological monotherapy in ameliorating affective–cognitive symptoms.

### 6.4 Mediating effect of sleep quality

The present study demonstrates that sleep quality mediates the effect of depressive symptoms on cognitive function. Specifically, robust associations emerged between depressive symptoms and sleep quality among rural older women. Sleep duration of fewer than 6 h correlates with depression in this population; sleep deprivation operates as a potential inflammatory promoter, heightening cellular inflammation and upregulating the transcription of interleukin-6 and tumor necrosis factor (46). Bidirectional associations between poor sleep and cognitive decline are well established in geriatric populations. Approximately 60–70% of older adults with cognitive impairment present with comorbid sleep disorders, and chronic insomnia in women confers a two-fold higher risk for incident dementia (47, 48). Employing chained mediation modeling, we further identified sleep disturbances as a partial mediator of the depressive symptoms–cognitive impairment link (indirect effect = −0.168, 95% CI: −0.239 to −0.101). These findings indicate that elevated depression severity exacerbates sleep disturbances, thereby compromising cognitive performance. Optimizing sleep hygiene facilitates nocturnal clearance of cerebral metabolic waste and restores immunological and endocrine homeostasis, consequently ameliorating depressive symptoms while attenuating cognitive deterioration in older adults.

## 7 Limitations

This investigation has several limitations warranting consideration. The cross-sectional design precludes causal inferences regarding the observed associations. Geographic specificity constitutes another constraint, as the data were derived exclusively from three southwestern Chinese regions, potentially compromising external validity beyond these locales. Third, reliance on self-reported instruments may introduce recall bias. Regarding recruitment, initial community announcements yielded incomplete participation; supplementary telephone invitations and door-to-door visits were subsequently employed. While this approach may have enriched the sample with highly motivated individuals, we anticipate minimal selection bias given the uniform eligibility criteria across recruitment modalities and comparable sociodemographic characteristics between participants and non-respondents.

Notably, the current analysis did not examine whether serial mediation effects vary across patient subgroups, precluding robustness assessments through stratified analyses. Furthermore, cognitive impairment prevalence exhibits substantial cross-national heterogeneity, which continues to shift in response to divergent environmental contexts and health policies. Future investigations incorporating longitudinal designs, broader geographic sampling, and objective biomarker assessments would elucidate the factors influencing physical and psychological well-being in aging populations.

## 8 Conclusion

This study of rural Chinese older women provides evidence that depressive symptoms are associated with cognitive function both directly and indirectly, with anxiety and poor sleep quality serving as partial mediators. These results highlight the importance of addressing psychological and sleep-related factors in understanding depression-related cognitive decline. Integrated treatment approaches—including cognitive-behavioral therapy, sleep hygiene programs, mindfulness training, physical activity, and evidence-based pharmacotherapy—may disrupt these detrimental pathways. Concurrent alleviation of depression, anxiety, and sleep disturbances could potentially preserve cognitive function and enhance well-being among rural older women.

## Data Availability

The raw data supporting the conclusions of this article will be made available by the authors, without undue reservation.
